# Host‐related factors and cancer: Malnutrition and non‐Hodgkin lymphoma

**DOI:** 10.1002/hon.3002

**Published:** 2022-04-18

**Authors:** Salvatrice Mancuso, Marta Mattana, Marco Santoro, Melania Carlisi, Silvio Buscemi, Sergio Siragusa

**Affiliations:** ^1^ Department of Health Promotion, Mother and Child Care, Internal Medicine and Medical Specialties (PROMISE), Hematology Unit, University of Palermo Palermo Italy; ^2^ Hematology Unit University Hospital “Paolo Giaccone” Palermo Italy

**Keywords:** cancer metabolic syndrome, diffuse large B‐cell lymphoma, frailty, nutritional status, non‐Hodgkin lymphoma

## Abstract

Assessment of host‐related factors is a crucial aspect in the comprehensive management of cancer patients. A distinct nutritional disturbance linked to cancer has been recognized to be associated with negative outcomes. However, compared to solid tumors, only a limited number of studies have looked specifically at nutritional issues in the field of lymphoma. The aim of this review is to integrate the current knowledge on interactions between malnutrition and lymphoma and address most relevant and pertinent questions. We first provide a literature review on the mutual biological relationship between malnutrition and lymphoma. Next, we explore the overlap between malnutrition, sarcopenia, cachexia and frailty in lymphoma studies. In addition, we summarize the clinical assessment scales used to measure malnutrition in lymphoma subjects. Furthermore, we address the problem of nutritional interventions aimed at patients who are candidates for treatment for lymphoma. Malnutrition can arise as a consequence of lymphoma disease and can in turn promote lymphomagenesis, negatively affect the response to therapy and favor adverse event to treatment. There is increasing evidence that malnutrition, sarcopenia and cachexia in lymphoma are intimately inter‐related and are a hallmark of frailty. A variety of different tools are recorded with the apparent ability to describe nutritional status and to impact prognosis in lymphoma patients. Finally, a network of prognostic host‐ and disease‐related factors is proposed where malnutrition can interact with each other in complex ways.

## 1 BACKGROUND

Lymphoma are a numerous group of biologically and clinically heterogeneous neoplastic entities, representing the 10th most common cancer and 11th leading cause of cancer deaths in the world, with a significant upward trend in the more advanced age groups.^1^, ^2^, ^3^, ^4^

Despite treatment advances, lymphoma remains a disease with limited treatment options for patients who relapse after prior therapy or with unfavorable prognostic disease or who failed to complete treatment due to adverse events.^5^, ^6^ In light of these findings, research needs to be expanded on the various factors that affect different subgroups of patients with lymphoma.

In general, host‐related factors appear to play a parallel and independent role with respect to tumor biology on clinical evolution of neoplastic disease.^7^ Indeed, as for lymphoma, the development of International Prognostic Index (IPI) and Revised International Prognostic Index (R‐IPI) arises from the need to predict outcome in patients with aggressive non‐Hodgkin's lymphoma (NHL) on the basis of the patient's clinical characteristics before treatment. This system incorporates clinical features that reflect the patient's response to the tumor (performance status), and the patient's ability to tolerate intensive therapy (age and performance status).^8^, ^9^

Furthermore, a complex interconnection network contributes to reciprocal effects between host‐related factors and the pathogenic aspects of the neoplasm. The outcome is therefore established by the final result of the converging effects of different variables related to both the host and the disease.^10^

In lymphoma some host‐tumor interactions are better characterized than others. The role of the microenvironment, host genetic factors, immunodeficiency, host infections or host exposure in the development of lymphoma and disease progression is known. The relative importance of these interactions appears to vary across lymphoma types.^11^, ^12^

Currently the information concerning the systemic interactions between lymphoma and the host, outside and at a distance from the neoplastic tissue, is fragmentary. However, the significant association between lymphoma onset and older age, for the biological implications that follow, provide an opportunity to analyze the role of degree of fitness and impairment of different organs and systems in lymphoma patients.^13^, ^14^

Among the systemic conditions that interact with the biology of lymphoma and are decisive for the progression of the disease, alterations in nutritional status represent a possible driver in the prognostic and evolutionary profile of lymphoma. The past years have seen an explosion of information regarding the role that dysregulated nutritional status plays in the etiology and progression of cancer.^15^, ^16^ Malnutrition in cancer patients is a topic strongly felt in the global cancer community to the point of being the subject of recommendations and guidelines.^17^, ^18^ With regard to hematological neoplasms and lymphoma, in particular, the issue has not yet sufficiently attracted general attention to the point of transferring a shared approach of malnutrition management into clinical practice. Furthermore, only few studies, have summarized the results obtained in this field.

This paper reviews the available evidence for a relationship between nutritional status and lymphoma. We focused on evaluating the following aspects: biological interactions between nutritional determinants and lymphomagenesis; correlation between nutritional diagnosis markers and clinical evolution of lymphoma; link between malnutrition, sarcopenia *e* frailty in lymphoma patients; exploration of nutritional models of intervention in lymphoma.

We performed a literature review between 2000 and 2021 using PubMed and Google Scholar, with the main focus on all articles addressing the topics listed above. Reference lists from previous reviews and key articles retrieved were also examined for relevant studies.

## 2 REVIEW

### 2.1 Definition of malnutrition

Malnutrition has been defined as a condition of an imbalance of energy, protein and other nutrients that can cause measurable negative effects on body composition, physical function and clinical outcomes. From the etiopathogenic point of view, it is necessary to consider various malnutrition syndromes that develop in specific clinical settings.^19^, ^20^ Using this approach, the role of the various risk factors is better defined. Cancers and their treatment can induce malnutrition by several mechanisms: in addition to inflammation, which constitutes the key contributor factor, a combination of varying degrees of reduced food intake and metabolic derangements leads to altered body composition and diminished biological function.^21^ In cancer patients, malnutrition significantly reduces response and tolerance to treatments, functional status, and quality of life. Consequently, nutritional status has been reported as a prognostic factor in patients with cancer.^22^ Malnutrition is frequent in hospitalized patients with lymphoma and is associated with higher mortality risk.^23^

### 2.2 Biological correlations between nutritional status and lymphoma

A growing body of evidence indicates that malnutrition is a cluster of conditions that can aggregate different levels of both undernutrition and obesity.^24^

Evaluation of previously specified lymphoma analyses will be important to know whether the findings in this setting differ from the overall field of cancer.

Some studies assessed the risk of onset of lymphoproliferative diseases in correlation with nutritional parameters.^25^, ^26^, ^27^, ^28^ In a nested case‐control study some markers of sustained B‐ cell activation were predictive of B‐cell lymphoma risk, namely sCD23, sCD27, sCD30, and CXCL13. Also, sCD23 and CXCL13 partly mediated the causal pathway association between positive Body Mass Index (BMI) and Diffuse Large B‐Cell Lymphoma (DLBCL) risk.^29^ Associations have also been reported between increased risk of NHL and polymorphisms in obesity‐related genes such as leptin (LEP) and leptin receptor,^2^, ^3^ key regulators of energy balance and immune function. Indeed, polymorphisms in the LEP gene (2548G>A, 19A>G), associated with high circulating leptin levels, were identified as susceptibility loci for NHL in two independent studies.^30^, ^31^

Obesity results in pathological states of low‐grade chronic inflammation with increased production of proinflammatory cytokines, such as interleukin‐6 (IL‐6), tumor necrosis factor‐*α*, interleukin‐ 1b (IL‐1b) and leptin. These cytokines can alter T‐ and B‐cell immune responses and enhance B‐cell proliferation and survival, both of which factors may promote lymphomagenesis.^32^

It is also acknowledged that undernutrition will further aggravate lymphoma evolution as well as promoting or causing new illness, as is the case for infections, which in the presence of malnutrition become more difficult to treat.

Epidemiological studies reinforce biological findings, suggesting an association between nutritional indicators and lymphoma risk and outcomes.^33^, ^34^ Indeed, several case‐control and prospective studies have found increased risk of NHL in association with BMI ≥ 30.^35^, ^36^, ^37^

Tumors actively perturb nutritional status through a variety of mechanisms. The anatomical site of presentation of the lymphoma, such as the gastrointestinal, oropharyngeal and central nervous system sites, can impair food intake. Beyond the mechanical causes that induce reduced intake, weight loss is a B‐symptom which can be found more frequently in high‐grade lymphoma or in those with faster replication. In cancer patients, important contributors of nutritional deterioration are represented by metabolic disturbance and changes in resting energy expenditure. Hypermetabolism and increased gluconeogenesis are not counterbalanced by adaptation mechanisms to preserve lean body mass. These deep metabolic disturbances are generated by a cascade of events triggered by biological mediators. The final outcome is that of a cancer metabolic syndrome.^38^ The intense production of pro‐inflammatory cytokines is attributable to the tumor itself or to the systemic response to the tumor. For lymphoma, a key clinical study highlighted the role of IL‐6 in causing anorexia and cachectic state that improved after treatment with anti‐IL‐6 monoclonal antibodies.^39^ Furthermore, depending on the treatment modality and on the type of regimen, therapy for lymphoma can impact nutritional status by means of side effects that accelerate the appearance of nutritional decline.^40^

### 2.3 Malnutrition, sarcopenia, cachexia and frailty in lymphoma

A multitude of concepts are reported in different studies to describe the host's condition with respect to neoplastic disease. In this regard, the clinical categories that are used are: undernutrition/overnutrition, malnutrition, sarcopenia and/or adipopenia, cachexia, frailty. First, we must consider that a general consensus does not exist on the term that best describes the nutritional depletion linked to cancer.

#### 2.3.1 Malnutrition and sarcopenia

The term sarcopenia refers to the loss of muscle mass.^41^ Initially restricted to the elderly population, over time it has gained a role in clinical evaluation in more diversified fields, especially in relation to tumors.^42^ In cancer patients, skeletal muscle protein degradation and breakdown as well decreasing protein synthesis are induced by cytokines through the alteration of various regulatory mechanisms. Therefore, a major role in triggering muscle protein damage is played by systemic inflammation. Inflammation is at the origin of nutritional and muscular damage and at the same time has an adverse effect on cancer outcome. In lymphoma, the action of cytokines produced by neoplastic cells in supporting a systemic inflammatory state has also been demonstrated.^43^, ^44^ Consequently, sarcopenia may reflect the biology of lymphoma. The prevalence of sarcopenia in NHL is reported between 55% and 58% of studied populations. Sarcopenia onset correlates to poor outcomes in elderly patients with diffuse large B‐cell lymphoma (DLBCL).^45^ However, in a retrospective study of 207 patients with DLBCL older than 18 years who received R‐CHOP, sarcopenia was proven to be an independent prognostic factor in male patients.^46^ The prognostic role of sarcopenia was assessed retrospectively in 187 patients with DLBCL. In this study sarcopenia was associated with an increased risk of chemotherapy toxicity and to poor prognosis.^47^ It would be necessary to evaluate the prognostic value of sarcopenia in large prospective studies. In addition, the study of sarcopenia today is part of a complex evaluation that concerns body composition metrics. Body composition is correlated with obesity, malnutrition, cachexia syndromes, metabolic syndrome and frailty. The preferred method for measuring and analyzing the whole‐body composition of patients is abdominal cross‐sectional imaging of computed tomography (CT) at the level of the third lumbar vertebra. In a recent retrospective study, Guo J et al. showed that measures of muscle metrics were more important than Body Surface Area (BSA) and BMI in predicting treatment toxicity and prognosis for DLBCL patients receiving R‐CHOP immunochemotherapy as initial treatment. The authors concluded that measurements of body composition obtained from conventional CT images may play a role in individualizing dosing regimens of antineoplastic drugs in the future.^48^ In another retrospective study, skeletal muscle radio‐density (SMD) on CT scans showed a strong link to follicular lymphoma response to immunochemotherapy regardless of Follicular Lymphoma International Prognostic Index (FLIPI).^49^ It has been proposed that lower SMD precedes the full development of sarcopenia and therefore is an earlier parameter in detecting muscle damage.

#### 2.3.2 Malnutrition and cachexia

Although consensus has been established for the definition and staging of cachexia,^19^, ^50^ across the different studies the terms cachexia and malnutrition sometimes acquire the same meaning, sometimes they are used to indicate similar but distinct conditions. In relation to solid tumors, cachexia is described as an epiphenomenon of end‐stage neoplasms. In lymphomas, the observations are not uniform. In a retrospective study, cachexia, when explored in the DLBCL by combining nutritional indices with sarcopenia, represented an independent prognostic variable with respect to National Comprehensive Cancer Network – IPI (NCCN – IPI).^51^ The same cannot be said for other studies, where, however, the inclusion criteria and characteristics of the populations were different. In a retrospective study, cachexia score including fat tissue loss (adipopenia) and sarcopenia as assessed by CT scan in elderly DLBCL treated with chemotherapy and rituximab was predictive of prognosis independent of BMI, IPI and albuminemia.^52^ Therefore, malnutrition describes a multitude of alterations that include numerous dysfunctional aspects. Indeed, in cancer patients, malnutrition is also closely related to functional status, muscle performance and quality of life. In a cross‐sectional observational study, a population of cancer patients with a significant prevalence of hematological neoplasms was evaluated. In this group, malnutrition positively correlated with Eastern Cooperative Oncology Group (ECOG)‐ performance status (PS) and negatively correlated with Karnofsky Performance Scale Index (KPSI) and Handgrip dynamometry.^16^

#### 2.3.3 Malnutrition and frailty

Malnutrition is part of a complex dysregulated system underlying frailty. Frailty is an age‐related syndrome characterized by weakness, weight loss, low activity and limited capacity to maintain homeostasis.^53^, ^54^ The diagnosis of frailty is predictive of adverse outcomes when exposure to stressors occurs. The cancer itself and chemotherapy can be source of stress along with the different causes of accumulating functional deficits.^55^ In lymphoma patients, frailty constitutes a complex clinical phenotype for which geriatric assessment is mandatory. With this approach, frailty appears more predictive of worse outcome in elderly lymphoma patients treated with R‐CHOP.^56^ Malnutrition and frailty are distinct entities that can arise independently as well as be causally related. However, the association between malnutrition and frailty and its impact on outcome of lymphoma patients remains underinvestigated.^57^

#### 2.3.4 Considerations

**FIGURE 1:**
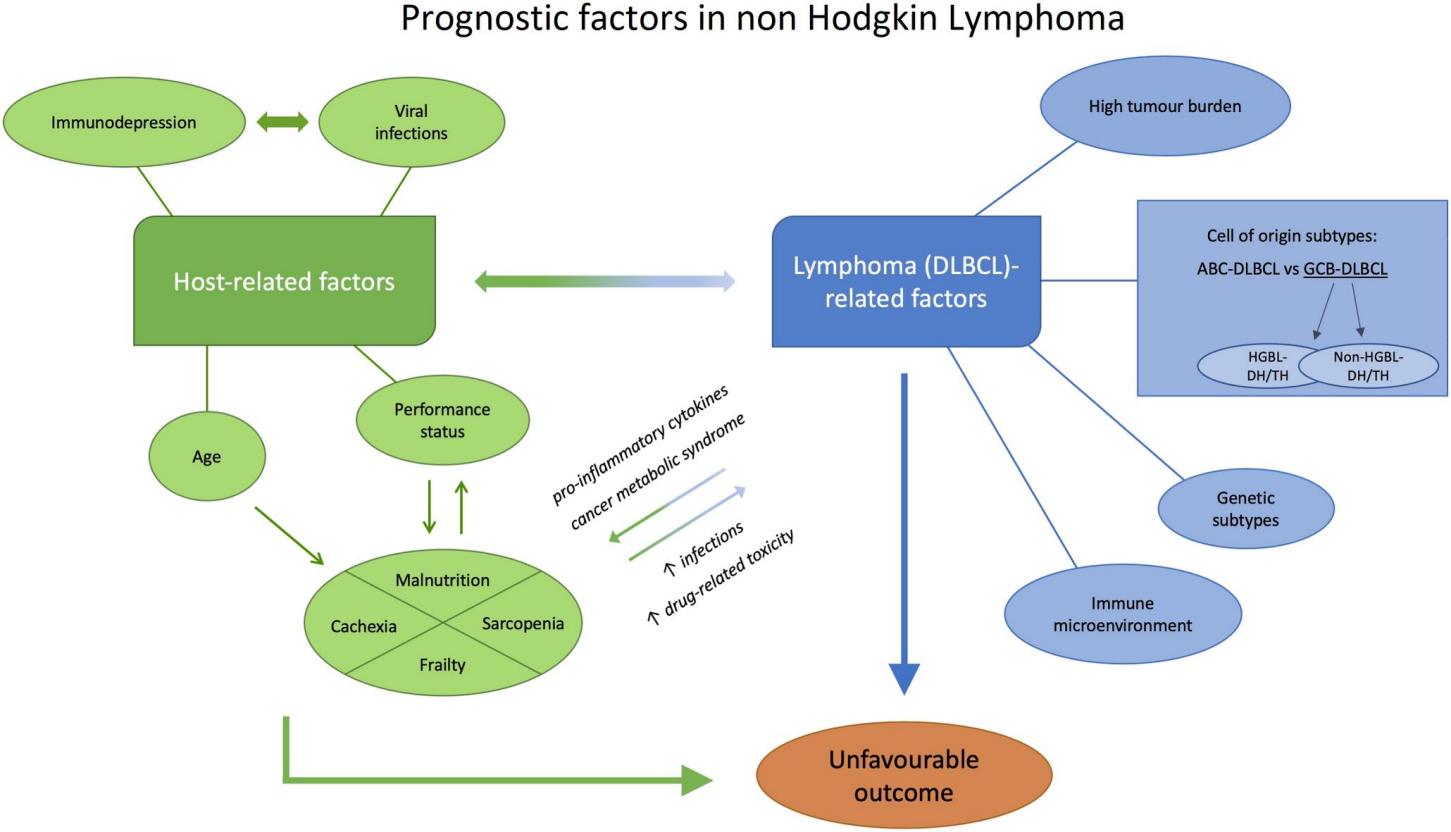
DLBCL, Diffuse Large B‐ cell Lymphoma; ABC‐DLBCL, Activated B‐Cell Like DLBCL; GCB‐DLBCL, Germinal Center B‐Cell Like DLBCL; HGBL‐DH/TH, High Grade Lymphoma‐ Double Hit/Triple Hit

Therefore, it may appear more appropriate to suggest that nutritional depletion in lymphoma patients is a complex clinical syndrome, characterized by the development of a number of different features. In this context, the specific contributions of changes in eating behavior, reduced food intake, weight loss, fatigue, muscle wasting, fat mass loss, impaired immune function, energy expenditure or intermediary metabolism largely vary from patient to patient. Although the molecular mechanisms responsible for this may share a number of common pathways, it may be useful to keep all the constituent elements of the syndrome separate in order to be able to establish the respective pathogenic contribution and identify possible targeted interventions.^58^ The main host and lymphoma factors that interact in determining the outcome of DLBCL are shown in Figure 1 (see).

### 2.4 Tools for the assessment of nutritional status and prognostic value in lymphomas

Malnutrition can impact disease progression and survival in cancer patients. Substantial studies have shown that weight loss in different types of cancer is associated with poor prognosis, poor Quality of Life (QoL), lower activity level, increased treatment‐related adverse symptoms, and reduced tumor response to therapy. However, the evaluation of nutritional status in clinical practice is considered complex and time‐consuming. Therefore, easy‐to‐apply methods have been generated by exploiting the study of some parameters.

Various specific tools have been developed to measure nutritional status as a prognostic factor of survival in cancer, taking into account the diversity of cancer types. At the moment, the ways to ascertain the nutritional status are the most varied and there is still no consensus on the best tool for nutritional assessment (see Table 1).

**TABLE 1:** Summary of studies of nutritional status in non‐Hodgkin lymphoma patients

Tools	Reference	Rituximab era	Study‐type	No. of patients	Patients age, y	Intervention	Clinical notes	Comments
BMI	^60^	Post‐rituximab	Retrospective	262	16–86	R‐CHOP	DLBCL	BMI was independently prognostic for OS in multivariate analysis
Albumin	^65^	Post‐rituximab	Retrospective	124	20–84	R‐CHOP	DLBCL	‐
	^61^	N.A.	Retrospective	68	<60 (48%) >60 (52%)	CHOP‐like regimes	PTCL‐NOS and AITL	Albumin was independent prognostic factor in PTCL‐NOS
	^64^	Post‐rituximab	Retrospective	39	16–72	CHOP/CHOP ‐like; R‐CHOP, EPOCH ± IF‐RT ± autologous HSCT	PMLBCL	Albumin predicted OS and PFS
	^63^	Post‐rituximab	Retrospective	309	33–86	No treatment, splenectomy,	SMZL	Albumin had negative prognostic influence on OS
			Multicenter			Immunotherapy, chemotherapy, interferon		
	^62^	N.A.	Retrospective Multicenter	136	<60 (70%)	CHOP‐like regimens	Peripheral T/NK lymphoma	Albumin prognostic factor in univariate analysis
	^66^	Post‐rituximab	Retrospective	157	18–90	R‐CHOP	DLBCL	Albumin was a strong prognostic factor for OS
ACA index	^82^	Post‐rituximab	Retrospective Multicenter	836	65–96	R‐CHOP	DLBCL	Albumin was included in an index score with age and comorbidities
CONUT score	^69^	Post‐ rituximab	Retrospective	476	27–97	R‐CHOP/R‐CHOP‐like	DLBCL	CONUT score was an independent prognostic score
PNI	^76^	Post‐rituximab	Meta‐analysis	1311	16–94	RCHOP/CHOP/R‐CVP, rituximab alone, palliative	DLBCL	Prognostic value of PNI on OS and PFS
							7 studies with Asian patients, 1 study with non‐Asian patients	
	^74^	Post‐rituximab	Retrospective	103	22–87	R‐CHOP/R‐CHOP like regimens	DLBCL	PNI was a predictor of response to treatment, OS and EFS
	^77^	Post‐rituximab	Retrospective	88	22–87	RT alone, chemotherapy alone, chemotherapy plus RT	FL	PNI was shown independent prognostic factor of PFS
	^75^	Post‐rituximab	Retrospective	228	21–88	R‐CHOP	DLBCL	PNI was associated with adverse clinical features
	^78^	N.A.	Retrospective Two centers	177	9–75	CHOP, CHOP/like, EPOCH/GEMOX	ENKTL	PNI was related to poor prognosis
	^73^	Post‐rituximab	Retrospective	253	19–81	R‐CHOP or CHOP		PNI was predictive for survival in patients treated with R‐CHOP; no effect in patients treated with CHOP
GNRI	^47^	Post‐rituximab	Retrospective	228	21–88	R‐CHOP	DLBCL	Negative prognostic factor in combination with sarcopenia
	^81^	Post‐rituximab	Retrospective Multicenter	615	20–97	R‐CHOP, R‐CHOP/like	DLBCL	GNRI also useful as prognostic factor for non GCB‐type DLBCL
	^80^	Post‐rituximab	Retrospective	476	27–97	R‐CHOP, R‐CHOP/like	DLBCL	GNRI had prognostic impact for OS and PFS
GPS	^83^	Post‐rituximab	Retrospective	252	16–82	R‐CHOP, CHOP	DLBCL	GPS was the most powerful indicator for survival compared to other inflammation based scores
	^87^	Post‐rituximab	Retrospective Multicenter	209	22–90	W&W	FL	GPS was the only independent predictor of OS and PFS in multivariate analyses
						CHOP‐like		
						Bendamustine		
						Rituximab‐based		
						Obinutuzumab‐base		
						RT		
						Anti‐CD20 ‐maintenance		

Abbreviations: AITL, Angioimmunoblastic T‐Cell Lymphoma; BMI, body mass index; COUNT, controlling nutritional status; DLBCL, Diffuse Large B‐Cell Lymphoma; EFS, event‐free survival; ENKTL, Extranodal NK/T‐cell lymphoma; EPOCH, etoposide, prednisone, vincristine, cyclophosphamide, doxorubicin; FL, Follicular Lymphoma; GNRI, geriatric nutritional risk index; GPS, Glasgow prognostic score; HSCT, hematopoietic stem cells transplantation; IF‐RT, involved field radiation therapy; N.A., not applicable; non‐GCB, non‐Germinal Center B‐cell; OS, overall survival; PFS, progression‐free survival; PMBCL, Primary Mediastinal B‐Cell Lymphoma; PTCL‐NOS, Peripheral T‐Cell Lymphoma, not otherwise specified; PNI, prognostic nutritional index; RT, radiation therapy; SMZL, Splenic Marginal Zone Lymphoma; W&W, watch and wait.

The significance of these parameters is to simultaneously describe the nutritional status and the inflammatory profile.

A Table describing how to use the tools reported in this text is available as Table S1 (see Table S2).

#### 2.4.1 Body Mass Index

Body Mass Index (BMI) is a simple anthropometric tool whose impact on the outcome of DLBCL is being studied in the immunochemotherapy era.^59^ In 262 patients with newly diagnosed DLBCL, BMI at diagnosis was independently prognostic for overall survival compared to other anthropometric and serological parameters: in particular, a BMI lower than 20 was the only variable associated with a better OS after multivariate analysis.^60^

#### 2.4.2 Albumin

The level of albumin at the moment appears the most studied nutritional parameter in a variety of contexts. It has a predictive role of outcome in a series of hematological neoplasms such as myelodysplastic syndromes, peripheral‐T cell lymphoma, splenic marginal zone lymphoma, primary mediastinal B‐cell lymphoma, Hodgkin lymphoma.^61^, ^62^, ^63^, ^64^ According to a retrospective study, serum albumin has been shown to be an independent prognostic marker in DLBCL patients treated with R‐CHOP as well as in those treated in the pre‐rituximab era.^65^ In another retrospective study, pretreatment albumin level has been shown to be a strong prognostic factor for Overall Survival (OS) in patients with DLBCL.^66^

#### 2.4.3 Controlling Nutritional Status Score

The Controlling Nutritional Status (CONUT) score, derived from serum albumin, absolute lymphocyte counts and cholesterol measurements, is an effective tool for assessing the status of immune nutrition.^67^, ^68^ In a retrospective study on 476 patients with DLBCL, CONUT score has been shown to be an independent poor prognostic factor of OS.^69^

#### 2.4.4 Prognostic Nutritional Index

Prognostic Nutritional Index (PNI) is a simple biomarker calculated on the basis of serum albumin level and total lymphocyte count in peripheral blood. It is used as prognostic parameter for various diseases, including solid tumors and hematological malignancies.^70^, ^71^, ^72^ Its prognostic value in lymphomas may reflect both the role of hypoalbuminemia and malnutrition in negatively affecting the outcome of the disease, both the intrinsic biological aggressiveness of lymphoma that induces immunosuppression as well as malnutrition through the action of cytokine mediators. In a retrospective study, PNI was identified as an independent predictor of response to treatment, OS and Event‐Free Survival (EFS) in patients with DLBCL.^73^ Furthermore, a significant correlation was observed between PNI and other poor prognostic factors including ECOG‐PS, bone marrow involvement, advanced disease stage and presence of B symptoms.^74^ In another retrospective study, low‐PNI was associated with more frequent therapy‐related toxicities and mortality and early withdrawal from treatment in patients treated with first‐line R‐CHOP for DLBCL.^75^ PNI also predicted both OS and EFS in a group of 98 patients with DLBCL in an additional retrospective study.^76^ The rationale behind PNI is also the basis of a retrospective study on a group of 88 patients with Follicular Lymphoma, in which baseline PNI played a significant role in independently predicting the outcome of disease.^77^ Finally, a retrospective study performed on 177 patients with extranodal natural killer/T cell lymphoma, nasal type, PNI was a powerful predictor of survival.^78^

A meta‐analysis work has recently examined how PNI is useful in predicting OS and Progression‐Free Survival (PFS) in patients with DLBCL. The aim of the analysis was to reach an unambiguous response in view of the conflicting results from previous studies. Data aggregation on 1311 DLBCL subjects showed that low PNI was a significant prognostic factor for poorer OS and poorer PFS. However, this investigation considered only seven small studies where patients were of Asian ethnicity only.^76^

#### 2.4.5 Geriatric Nutritional Risk Index

The Geriatric Nutritional Risk Index (GNRI) was developed as a simple method to assess nutritional status which utilizes three objective parameters: body weight, height and serum albumin. It was originally designed for the assessment of the risk associated with malnutrition in elderly medical patients.^79^

The role of the GNRI in predicting clinical outcomes of diffuse large B cell lymphoma was proven in a total of 476 retrospectively analyzed patients with newly diagnosed de novo DLBC.^80^ In a retrospective study on 227 DLBCL patients, a combined score of GNRI and sarcopenia revealed two groups at different prognosis: high cachexia risk group with a lower complete response rate to R‐CHOP, higher frequency of treatment‐related mortality and early treatment discontinuation compared to low cachexia risk group.^51^

In a multicenter retrospective study, the prognostic values of GNRI, PNI and CONUT were compared across 615 newly diagnosed DLBCL patients. In multivariate analyses, baseline poor nutritional status determined by GNRI or CONUT was an independent risk factor of newly diagnosed DLBCL. Moreover, GNRI was also useful as an independent prognostic factor for patients with non‐ Germinal Center B‐cell like (GCB)‐type DLBCL.^81^

#### 2.4.6 ACA Index

Albumin, along with age and comorbidities, is included in another score known as the Age, Comorbidities, and Albumin (ACA) index, which was developed and validated in a study on elderly DLBCL patients. In this group, the ACA index was found to be useful as a predictor of prognosis, tolerability to cytotoxic drugs, and adherence to R‐CHOP treatment.^82^

#### 2.4.7 Glasgow Prognostic Score

Based on the assumption that nutritional status constitutes a counterpart to the host inflammatory response, we report a retrospective study on 252 DLBCL patients treated with R‐CHOP that explored the prognostic significance of different inflammation‐base scores. The Glasgow Prognostic Score (GPS) was the most powerful marker in predicting survival when compared to prognostic index (PI), PNI, neutrophil lymphocyte ratio (NLR) and platelet‐lymphocyte ratio (PLR).^83^ Based on the concentration of C‐reactive protein and albumin, GPS has been derived from patients with various types of cancer and validated in multiple cohorts.^84^, ^85^, ^86^ Finally, in 209 patients with FL, a retrospective multicenter study showed prognostic impact of GPS on OS and PFS.^87^

## 3 NUTRITIONAL INTERVENTIONS

Nutritional problems in cancer patients are widely recognized as well as possible strategies to rebalance malnutrition during antineoplastic treatment.^18^, ^88^ There is awareness that in addition to malnutrition at the time of diagnosis caused by the metabolic effects of the tumor, common side effects of cancer treatment can lead to inadequate nutrient intake and further worsening of the nutritional state. However, when looking at specific types of hematological tumors, we find few interventional studies.

As regards hematological neoplasms, nutritional status is at the center of growing interest in the context of chronic pathologies. In myeloproliferative neoplasms (MPN), chronic inflammation plays a major role in disease progression and in sustaining a high symptom burden. Nutritional interventions are among the non‐pharmacological treatments that are being examined to modulate the inflammatory state and improve the systemic symptoms. To date, preliminary feasibility studies of nutritional interventions on MPN patients are available.^89^

Another area of interest concerns nutritional support in patients with hematological malignancies undergoing bone marrow transplantation.^90^, ^91^ Therefore, as yet, no data and experiences have been published on the application of models of nutritional intervention in patients with lymphoma and in particular with DLBCL. Consequently, reference is made to the recommendations relating to oncology in general.

Finally, we must remember that the Covid‐19 pandemic has put the management of routine health needs in crisis. In this challenging period, telemedicine has become crucial in mitigating the negative impact of the pandemic in cancer patients. A simple remote nutritional tool, Remote‐Malnutrition Application for Primary Practice (R‐MAPP), has been proposed to recognize and manage nutritional needs related to a number of diseases including neoplasms. This tool explores nutritional risks and sarcopenia through Malnutritional Universal Screening Tool (MUST) and Sarc‐F (5‐item questionnaire) performed remotely. This‐type of approach involves also nutritional therapy tailored according to the patient's needs.^91^ Therefore, due to the new capability of telemedicine in reconfiguring the clinical practice, this type of intervention can represent a cost‐effective solution to achieve the nutritional control of patients with lymphoma.^92^

## 4 CONCLUSIONS AND FUTURE PERSPECTIVE

Extensive studies on the outcomes of NHL, and in particular of DLBCL, have focused on the cellular and molecular network which underlies resistance to treatments and disease progression. In contrast, little is known about the complex host‐related phenomena that generate vulnerability to disease and treatments, worsening in turn the prognosis.^93^

While the recent years have seen a growth of interest on the role that dysregulated nutritional status plays in the etiology and progression of cancer, the data limited to the specific field of lymphomas at the moment are to be considered preliminary.

Some of the cellular and molecular mechanisms underlying the effects of altered nutritional control systems on lymphomagenesis have been described. In a likely landscape, B‐cell activation is the putative mediator between the involvement of genes responsible for nutritional control and onset and progression of lymphoma. The lymphoma in turn triggers a chain of pathological and inflammatory events which induce a metabolic breakdown resulting in malnutrition.

Interactions between lymphoma biology and host‐related factors create a vicious circle. Due to an intricate web of interrelationships, malnutrition, sarcopenia and frailty can be consequence and expression as well as partly responsible for the biological characteristics of the neoplasm.

In this regard, the malnutrition state acts in synergy with the aggressiveness of the lymphoma in increasing the risk of treatment failure.

Despite these premises, only observational retrospective studies were carried out, resulting in a very limited production of new clinical data to be rapidly implemented into the care process. In the reported studies, it is noted that significant differences arise between tools applied as biomarkers of malnutrition in the various series of lymphoma patients. There is no clear distinction between biomarkers that are well established from those that are used merely for research purpose. Furthermore, to improve generalizability, it is desirable that future studies be more inclusive compared to those published to date as regards the ethnicity of the patients enrolled. Despite our comprehensive search for interventional studies on this topic, we didn't find interventional trials—a sobering fact that underscores the need for further work in this area.

What emerges from the analysis of the available evidence is the complexity and diversity of all the aspects related to nutritional status in lymphoma settings that the research community is attempting to explore.

At the moment, it seems premature to foresee having recommendations and guidelines that support the integration of the nutritional study in the prognostic assessment at the time of lymphoma diagnosis, allowing a multi‐specialistic as well as personalized approach.

As integrative hematological oncology continues to evolve and expand, nutritional features must be standardized to accurately guide clinicians and researchers.

Advancing research in this area will be an opportunity for cross‐disciplinary interaction and knowledge exchange. On the basis of these premises, a perspective new interdisciplinary “onconutritional” science is emerging.

## CONFLICT OF INTEREST

The authors have no relevant financial or non‐financial interests to disclose.

## ETHICAL APPROVAL

This article does not contain any studies with human participants or animals performed by any of the authors.

## AUTHOR CONTRIBUTIONS

Salvatrice Mancuso conceived and wrote the review, Marta Mattana performed the literature search, Marco Santoro and Melania Carlisi reviewed and edited the manuscript, Marco Santoro submitted the article, Silvio Buscemi and Sergio Siragusa overviewed and critically revised the work.

### PEER REVIEW

The peer review history for this article is available at https://publons.com/publon/10.1002/hon.3002.

## Supporting information

Supplementary Material S1

Supplementary Material S2

## Data Availability

Data sharing not applicable to this article as no datasets were generated or analyzed during the current study.
