# Supplementary material for: Host‐related factors and cancer: Malnutrition and non‐Hodgkin lymphoma
Source: Hematol Oncol. 2022 Apr 18;40(3):320–31. doi: 10.1002/hon.3002 (PMC9544175; doi:10.1002/hon.3002)
Supplement: Supplementary file 1 — Supplementary Material S1 [file HON-40-320-s001.docx]

**Supplementary material 1.** CONUT score: *Controlling Nutritional Status score*; PNI: *Prognostic Nutritional Index*; GNRI: *Geriatric Nutritional Risk Index*; ACA: *Age, Comorbidities and Albumin*; GPS: *Glasgow Prognostic Score*.
