# Supplementary material for: Host‐related factors and cancer: Malnutrition and non‐Hodgkin lymphoma
Source: Hematol Oncol. 2022 Apr 18;40(3):320–31. doi: 10.1002/hon.3002 (PMC9544175; doi:10.1002/hon.3002)
Supplement: Supplementary file 2 — Supplementary Material S2 [file HON-40-320-s002.pdf]

								Interpretation
**CONUT score**	points	0	1	2	3	4	6	Undernutrition degree
	Serum Albumin (g/dl)	*≥ 3.50*		*3.00-3.49*		*2.50-2.99*	*<2.50*	0-1 = Normal<br>2-4 = Light
	Absolute Lymphocyte Count (#/mmc)	*≥ 1600*	*1200-1599*	*800-1199*	*<800*			5-8 = Moderate
	Total Cholesterol (mg/dl)	*≥ 180*	*140-179*	*100-139*	*<100*			9-12 = Severe
**PNI**	10 × *serum albumin (g/dl)* + 0.005 × *total lymphocyte count (#/mm3)*							≥ 50 = Normal<br>< 50 = Mild malnutrition<br>< 45 = Moderate-severe malnutrition<br>< 40 = Serious malnutrition.
**GNRI**	1.489 x *serum albumin (g/l)* + 41.7 x *(weight in kilograms/ideal weight)*							Nutrition-related risk:<br>< 82 = Major risk<br>82 - 91 = Moderate risk<br>92 - 98 = Low risk<br>> 98 = No risk
**ACA index**	points	0	1					0 points = Excellent
	Age	*≤ 74*	*> 75*					1 point = Good
	Serum Albumin (g/dl)	*≥ 3.8*	*< 3.7*					2 points = Moderate
	Charlson Comorbidity Index	*< 3*	*≥ 3.1*					3 points = Poor
**GPS**	points	0	1					0 points = Good
	C reactive protein (mg/lt)	*≤ 0.5*	*> 0.5*					1 point = Intermediate
	Serum Albumin (g/dl)	*≥ 3.5*	*< 3.5*					2 points = Poor
